# A 3-Year-Old Girl with Recurrent Infections and Autoimmunity due to a *STAT1* Gain-of-Function Mutation: The Expanding Clinical Presentation of Primary Immunodeficiencies

**DOI:** 10.3389/fped.2017.00055

**Published:** 2017-03-17

**Authors:** Juan Carlos Aldave Becerra, Enrique Cachay Rojas

**Affiliations:** ^1^Allergy and Immunology Division, Hospital Nacional Edgardo Rebagliati Martins, Lima, Peru

**Keywords:** signal transducer and activator of transcription 1, gain-of-function mutation, immunodeficiency, autoimmunity, hematopoietic stem cell transplantation

## Abstract

We report a 3-year-old Peruvian girl, born to non-consanguineous parents. At the age of 8 months, she had a severe pneumonia complicated with empyema that required thoracic drainage and mechanical ventilation. Although no microorganisms were isolated, the patient recovered with broad-spectrum antibiotics. Since that date, she has presented multiple episodes of pneumonia and recurrent episodes of bronchospasm. At 1 year 5 months of age, the patient began with recurrent episodes of oropharyngeal, vaginal, and skin candidiasis, which improved transiently after using oral azole drugs. At 2.5 years of age, she was admitted with lupus-like syndrome, including serositis, hemolytic anemia, thrombocytopenia, and positive antinuclear (1:80) and dsDNA (1:10) autoantibodies. Available immunologic testing was not contributory. Imaging studies revealed bilateral ethmoidal sinusitis and mild hepatomegaly. Bone marrow analysis did not showed evidence of leukemia or myelodysplasia, while renal biopsy concluded mild mesangial proliferation. Genetic studies revealed a pathogenic heterozygous *signal transducer and activator of transcription 1* gain-of-function mutation (WT/P293L). The clinical status and lung function of the patient has worsened progressively. She has not achieved an optimal response to therapy, including high-dose intravenous immunoglobulin, GM-CSF, prophylactic antibiotics and antifungal drugs, so we plan to perform hematopoietic stem cell transplantation.

## Introduction and Background

We report the case of a 3-year-old Peruvian girl who displayed recurrent infections by *Candida albicans* and pyogenic bacteria, as well as clinical features of autoimmunity. Our patient was born to non-consanguineous Mestizo parents. There was no family history suggestive of primary immunodeficiencies (PIDs). Her parents and an older brother were apparently healthy. The patient had no adverse reaction to the BCG vaccine.

We evaluated the patient for the first time when she was 2.5 years old. She had been admitted to our hospital with a diagnosis of lupus-like syndrome, including serositis (pleural and pericardial effusion), hemolytic anemia, autoimmune thrombocytopenia, and proteinuria. Laboratory testing revealed the presence of positive antinuclear (1:80) and dsDNA (1:10) autoantibodies. Imaging studies reported bilateral ethmoidal sinusitis and mild hepatomegaly. Bone marrow smear and biopsy did not show evidence of leukemia or myelodysplasia, while renal biopsy concluded the existence of mild mesangial proliferation. At that time, clinical disease partially improved with systemic corticosteroids.

After looking at her past clinical history, we realized that the patient also displayed increased susceptibility to infections. At the age of 8 months, she had a severe pneumonia complicated with empyema that required thoracic drainage and mechanical ventilation. Although no microorganisms were isolated, the patient recovered with broad-spectrum antibiotics. From then, she had multiple episodes of pneumonia and recurrent bronchospasm that required long-term treatment with inhaled corticosteroids. Furthermore, since 1 year 5 months of age, the patient suffered from recurrent oropharyngeal, vaginal, and skin infections by *C. albicans*, which worsened while receiving corticosteroids for the autoimmune phenomena and improved transiently after using oral fluconazole. Chronic diarrhea and failure to thrive were prominent clinical features from 2 years of age.

Upon suspicion of PID, available immunologic work up was requested. Serum immunoglobulins were within normal levels, as well as T, B, and NK lymphocyte counts. Serum complement proteins C3, C4, C1, C1q, and C2 were not decreased.

Advanced immunologic testing, including lymphoproliferation studies, assessment of T helper subpopulations, and innate immune functional evaluation were not available in Peru at that time. Therefore, we quickly decided to search for international collaboration. Two months later, genetic studies using Sanger sequencing revealed a heterozygous gain-of-function (GOF) mutation (P293L) located in the coiled-coil domain of the transcription factor *signal transducer and activator of transcription 1 (STAT1)*.

Despite therapy, the clinical status and lung function of the patient has worsened progressively over the following months. Computer tomography of the chest at 3 years of age revealed diffuse chronic inflammatory lung disease with bilateral bronchiectasis. The patient has not achieved successful response to several treatments, including prophylactic cotrimoxazole, azithromycin and fluconazole, high-dose (1 g/kg monthly) intravenous immunoglobulin, and granulocyte colony-stimulating factor (G-CSF). Therefore, we consider her a candidate to receive hematopoietic stem cell transplantation (HSCT).

However, there are several limitations to perform HSCT in developing countries like Peru. These limitations include (a) lack of bone marrow national registry to find HLA-matched unrelated donors, (b) difficulty to carry out haploidentical HSCT, and (c) scarce human, technologic, administrative, and economic resources. Our reported patient does not have a HLA-matched related donor. As a consequence, 3 years after performing the definitive genetic diagnosis, HSCT has not been done yet. It is important to consider that efficacy of HCST to treat severe disease due to *STAT1* GOF mutations has not been fully defined.

## Discussion

*Signal transducer and activator of transcription 1* GOF mutations reduce the dephosphorylation of activated STAT1 protein, leading to accumulation of phosphorylated STAT1 in the nucleus (1, 2). Persistently activated STAT1 may shift the immune response toward STAT1-dependent interleukin-17 inhibitors and away from STAT3-mediated TH17 cell generation (3, 4). GOF mutations affecting *STAT1* lead to defective TH17 cell development, characterized by reduced production of IL-17 and IL-22; these cytokines are crucial for antifungal and antibacterial defense in skin and mucosa (2, 5–8). An increased signaling of interferons and IL-27 through STAT1 cause an elevated risk of autoimmune phenomena (2).

Patients with GOF *STAT1* mutations usually present with autosomal dominant chronic mucocutaneous candidiasis and infections by extracellular bacteria, mainly *Staphylococcus aureus* (2). Other prominent clinical manifestations include cutaneous dermatophytosis, cerebral aneurisms, carcinomas, mycobacterial disease, and autoimmune phenomena such as hypothyroidism, autoimmune hepatitis and lupus-like syndrome (3, 6, 9). GOF *STAT1* mutations can also underlie disseminated coccidioidomycosis and histoplasmosis (10), recalcitrant cutaneous fusariosis (11), susceptibility to viral infections (12, 13), IPEX-like syndrome (14), severe combined immunodeficiency (15), and common variable immunodeficiency (16).

Current therapy for patients with GOF *STAT1* mutations is based on the use of long-term antifungal and antibacterial drugs (2). Some affected individuals have been treated with the JAK1/JAK2 inhibitor ruxolitinib (17, 18), G-CSF, GM-CSF, and intravenous immunoglobulin (9), with inconsistent outcomes. HSCT should be considered as a treatment option for patients with severe clinical course (9). Availability of HSCT might be a critical determinant of survival in these patients.

## Concluding Remarks

We report a 3-year-old Peruvian girl with a GOF *STAT1* mutation who displayed infections by *C. albicans* and pyogenic bacteria, as well as clinical features of autoimmunity. The clinical presentation of GOF *STAT1* mutations is highly variable. Severe life-threatening disease might require HSCT, a complex therapy that usually is not promptly available in developing countries.

## Informed Consent

Parents of the patient gave written informed consent to publish the current manuscript in accordance with the Declaration of Helsinki.

## Author Contributions

JB and ER contributed equally to the elaboration of the manuscript.

## Conflict of Interest Statement

The authors declare that the research was conducted in the absence of any commercial or financial relationships that could be construed as a potential conflict of interest.
